# Neurotoxic Microglial Activation via IFNγ‐Induced Nrf2 Reduction Exacerbating Alzheimer's Disease

**DOI:** 10.1002/advs.202304357

**Published:** 2024-03-14

**Authors:** You Jung Kang, Seung Jae Hyeon, Amanda McQuade, Jiwoon Lim, Seung Hyun Baek, Yen N. Diep, Khanh V. Do, Yeji Jeon, Dong‐Gyu Jo, C. Justin Lee, Mathew Blurton‐Jones, Hoon Ryu, Hansang Cho

**Affiliations:** ^1^ Institute of Quantum Biophysics Sungkyunkwan University Suwon Gyeonggi 16419 Republic of Korea; ^2^ Department of Biophysics Sungkyunkwan University Suwon Gyeonggi 16419 Republic of Korea; ^3^ Center for Brain Disorders Brain Science Institute Korea Institute of Science and Technology Seoul 02792 Republic of Korea; ^4^ Institute for Neurodegenerative Diseases University of California San Francisco CA 94158 USA; ^5^ Department of Neurobiology & Behavior University of California Irvine Irvine CA 92697 USA; ^6^ Sue and Bill Gross Stem Cell Research Center University of California Irvine Irvine CA 92697 USA; ^7^ Institute for Memory Impairments and Neurological Disorders University of California Irvine Irvine CA 92697 USA; ^8^ IBS School University of Science and Technology (UST) Daejeon 34114 Republic of Korea; ^9^ Center for Cognition and Sociality Institute for Basic Science (IBS) Daejeon 34126 Republic of Korea; ^10^ School of Pharmacy Sungkyunkwan University Suwon Gyeonggi 16419 Republic of Korea; ^11^ Department of Intelligent Precision Healthcare Convergence Sungkyunkwan University Suwon Gyeonggi 16419 Republic of Korea; ^12^ Biomedical Institute for Convergence Sungkyunkwan University Suwon Gyeonggi 16419 Republic of Korea; ^13^ Samsung Advanced Institute for Health Sciences and Technology Sungkyunkwan University Seoul 16419 Republic of Korea

**Keywords:** Alzheimer's diseases, interferon‐gamma, microglia, neurodegeneration, neuroinflammation, oxidative stress

## Abstract

Microglial neuroinflammation appears to be neuroprotective in the early pathological stage, yet neurotoxic, which often precedes neurodegeneration in Alzheimer's disease (AD). However, it remains unclear how the microglial activities transit to the neurotoxic state during AD progression, due to complex neuron‐glia interactions. Here, the mechanism of detrimental microgliosis in AD by employing 3D human AD mini‐brains, brain tissues of AD patients, and 5XFAD mice is explored. In the human and animal AD models, amyloid‐beta (Aβ)‐overexpressing neurons and reactive astrocytes produce interferon‐gamma (IFNγ) and excessive oxidative stress. IFNγ results in the downregulation of mitogen‐activated protein kinase (MAPK) and the upregulation of Kelch‐like ECH‐associated Protein 1 (Keap1) in microglia, which inactivate nuclear factor erythroid‐2‐related factor 2 (Nrf2) and sensitize microglia to the oxidative stress and induces a proinflammatory microglia via nuclear factor kappa B (NFκB)‐axis. The proinflammatory microglia in turn produce neurotoxic nitric oxide and proinflammatory mediators exacerbating synaptic impairment, phosphorylated‐tau accumulation, and discernable neuronal loss. Interestingly, recovering Nrf2 in the microglia prevents the activation of proinflammatory microglia and significantly blocks the tauopathy in AD minibrains. Taken together, it is envisioned that IFNγ‐driven Nrf2 downregulation in microglia as a key target to ameliorate AD pathology.

## Graphical abstract

Mechanisms of detrimental microgliosis in Alzheimer's disease are explored. Interferon‐gamma (IFNγ) upregulates Kelch‐like ECH‐associated Protein 1 (Keap1) in microglia, which inactivates nuclear factor erythroid‐2‐related factor 2 (Nrf2) and sensitizes microglia to the oxidative stress, resulting in the transition of proinflammatory microglia via nuclear factor kappa B (NFκB)‐axis. The proinflammatory microglia in tern exacerbate synaptic impairment, phosphorylated‐tau accumulation, and decernable neuronal loss at the end.

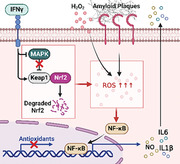

## 1 Introduction

Alzheimer's disease (AD), the most common cause of dementia, typically involves neuronal damage followed by cognitive decline in aging brains, which impact the lives of million people in the world.^[^ ^1^ ^]^ Key signatures of AD include deposition of neuritic amyloid‐beta (Aβ) plaques and neurofibrillary tau tangles (NFTs) leading to synaptic impairment and neuronal death.^[^ ^2^ ^]^ In addition, prominent activation of innate immune cells followed by neuroinflammation have been observed through the AD progression.^[^ ^3^ ^]^ Recent studies have revealed that the neuroinflammation is the major factor to proceed AD pathology by promotion of tauopathy, elevation of neurotoxic inflammatory mediator levels, and decrease of neurotransmitter levels.^[^ ^4^ ^]^ Such consequences of the neuroinflammation further result in synaptic and cognitive impairment, neuronal death, reduced memory, and brain dysfunctions within the neocortex and hippocampus.^[^ ^5^ ^]^ In addition, the degree of inflammatory cell activation and cytokine production can correlate with the severity of clinical symptoms of AD.^[^ ^6^ ^]^

Among the innate immune cells governing the neuroinflammation, microglia, brain‐resident myeloid cells, offer immune surveillance of the central nervous system (CNS) and play neuroprotective roles by removing excessive Aβ, tau oligomers, and other cellular debris.^[^ ^7^ ^]^ Microglia also provide neuroprotection by secreting anti‐inflammatory cytokines, growth factors, and neurotrophic factors.^[^ ^8^ ^]^ Recent studies identified unique disease‐associated microglia (DAM) types, serving effective phagocytosis and clearance of pathological aggregates, particularly Aβ plaques.^[^ ^9^ ^]^ Due to their pivotal roles, dysregulation of microglial functions can on the other hand promote Aβ and tau accumulation, further driving the development and progression of AD.^[^ ^9^, ^10^ ^]^ Numerous studies have reported other detrimental roles of microglia in neurodegenerative brains, such as the adoption of proinflammatory states and exacerbated synaptic loss followed by neuronal death.^[^ ^4^, ^11^ ^]^ However, it remains unclear how microglia transition from a more neuroprotective state to more detrimental states in part because of the lack of appropriate human microglial models to investigate and identify the key mediators that underlie this transition in the context of AD pathogenesis.

Herein, we employed our 3D human AD mini‐brains with human induced pluripotent cell (hiPSC)‐derived microglia (iMG) to clarify stage‐specific microglial phenotypes and identified key modulators driving the phenotype transition during AD progression. Our human AD mini‐brains closely reproduced key representative AD features of pathological accumulation of Aβ, phosphorylated tau accumulation, and glial proinflammation. In addition, our platform enabled the separation of activated subset of microglia by chemotaxis of AD cues and the identification of key modulators driving the phenotype transition in AD progression. Finally, we validated the underlying mechanisms of stage‐specific microgliosis by using tissue samples from human AD patients and 5XFAD mice.

## 2 Results

### 2.1 Construction of Pathophysiological Human AD Minibrains with hiPSC Microglia

**Figure 1:**
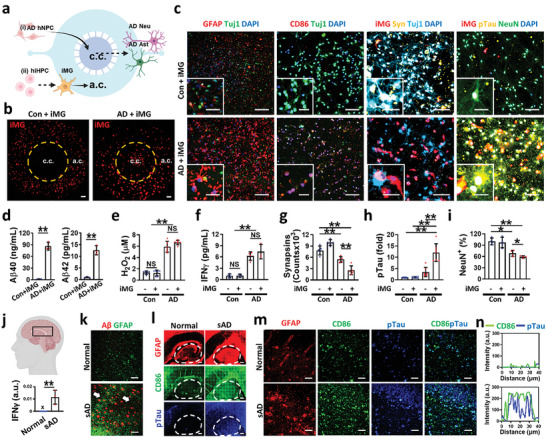
Neurodegeneration in IFNγ‐releasing 3D human AD mini‐brains and brain tissues of AD patients. a) Schema of 3D human AD minibrain construction. i) AD hNPCs overexpressing APPSL/PSEN1 were cultured and differentiated into Aβ‐overproducing neurons (AD Neu) and astrocytes (AD Ast) in the central compartment (c.c). ii) Microglia (iMG) differentiated from hiPSC‐derived hiHPCs were seeded in the angular compartment (a.c). b) Live‐cell images showing the microglia migration toward the central chamber in response to soluble factors from the 3D cultured human neurons and astrocytes in AD minibrains (AD+iMG) while no response in control brains (Con+iMG). c) Immunofluorescent images confirming astrogliosis (GFAP), microgliosis (CD86), synaptic impairment (Syn), and Tau phosphorylation (pTau) in AD mini‐brains. d–f) AD pathological evidence of d) increased Aβ_1‐40_ (Aβ40, two‐tailed unpaired *t*‐test, *n* = 3) and Aβ_1‐42_ (Aβ42, two‐tailed unpaired *t*‐test, *n* = 3), e) H_2_O_2_ (one‐way ANOVA with Tukey's multiple‐comparisons test, *n* = 4), and f) IFNγ (one‐way ANOVA with Tukey's multiple‐comparisons test, *n* = 5) in human AD minibrains. g–i) Assessment of neurodegeneration with g) reduced synapsin expression (one‐way ANOVA with Tukey's multiple‐comparisons test, *n* = 5), h) accumulated pTau (one‐way ANOVA with Tukey's multiple‐comparisons test, *n* = 7), and i) diminished neuronal populations in human AD minibrains (one‐way ANOVA with Tukey's multiple‐comparisons test, *n* = 4). j,k) Hippocampal regions of severe AD patient tissues (sAD) showing j) the increased IFNγ level confirmed by qPCR (two‐tailed unpaired *t*‐test, *n* = 4) and k) Aβ plaques (white arrows) by immunostaining compared to healthy individuals (Normal). l,m) Fluorescent images of l) hippocampal and m) CA1 regions confirming astrogliosis (GFAP), microgliosis (CD86), and Tau phosphorylation (pTau). n) Presence of neurodegenerative microglia (green) near pTau (blue) in severe sAD. All data represents means ± SD. “X,” under the limit of detection; “NS,” non‐significance; *, *P* < 0.05, **, *P* < 0.01. Scale bars, b) 500 µm, c) 300 µm and 100 µm in inset, k) 100 µm, l) 1 mm, m) 40 µm.

We recently created 3D human AD minibrains, which closely mimicked neuron‐glia signatures found in AD by culturing human AD neurons, astrocytes, and microglia in chemotactic microfluidic platforms.^[^ ^4^, ^10^, ^12^ ^]^ Here, we employed our versatile AD mini‐brains to investigate the phenotypes of hiPSC‐derived microglia (iMG) or human adult microglia (MG) in AD progression. Briefly, human neuroprogenitor cells (hNPCs) expressing familial AD (FAD) mutations (AD hNPCs) were cultured in the 3D central microchamber (c.c.) and incubated under 2 weeks of serum starvation to induce differentiation into AD neurons and astrocytes (**Figure** **1**a and Figure S1a, Supporting Information).^[^ ^13^ ^]^ For the control counterpart, we seeded the hNPCs expressing only GFP (Control hNPCs). After 2 weeks of serum starvation, both mini‐brains were incubated in the same condition for additional 6–9 weeks to reconstitute a more mature AD microenvironment.^[^ ^4^ ^]^ We refer the cocultured AD neurons and astrocytes as “AD” and the counter part as “Con.” Afterward, iMGs derived using a well‐established method,^[^ ^10^, ^14^ ^]^ were seeded into the angular chamber (a.c.) (Figure 1a and Figure S1b, Supporting Information). This angular chamber was connected to the central chamber via migration channels, which formed gradients of soluble factors produced by neurons and astrocytes within the central chamber, so that microglia were recruited to the central chamber of AD cultures (AD+iMG), but not Con cultures (Con+iMG), within 2 d (Figure 1b). In this way, we could engage active microglia only in response to AD soluble factors out of heterogeneous populations.^[^ ^4^, ^10^, ^15^ ^]^

To evaluate our AD models, we assessed major AD hallmarks within AD mini‐brains (Figure 1c–i) and compared them to hippocampal samples from AD patients (Figure 1j–n and Table S1, Supporting Information), and conventional AD animal models involving same FAD mutations as APP/PS1 mice (Figure S2, Supporting Information). We confirmed the presence of Aβ signatures including Aβ40 (86.3 ng mL^−1^), Aβ42 (12.6 pg mL^−1^) (Figure 1d),^[^ ^16^ ^]^ and Aβ plaques (Figure 1k and Figure S2a, Supporting Information).^[^ ^12^, ^17^ ^]^ In response to amyloid deposition, glial activity was increased in AD conditions; including activated astrocytes and microglia as detected by increased glial fibrillary acidic protein (GFAP)^[^ ^4^, ^18^ ^]^ and cluster of differentiation 86 (CD86) expression,^[^ ^19^ ^]^ respectively (Figure 1c,l,m and Figure S2a,b, Supporting Information). In addition, we detected significant increase of proinflammatory mediators including hydrogen peroxide (H_2_O_2_) (Figure 1e) and interferon‐gamma (IFNγ) in AD mini‐brains (Figure 1f) as well as AD patient brains (Figure 1j), which would be produced by GFAP‐positive reactive astrocytes, co‐cultured with AD neurons (Figures S3a and S4, Supporting Information).^[^ ^4b,c^ ^]^ As a consequence of glial activity, AD models exhibited signs of neurodegeneration including decreased presynaptic terminals (Figure 1g), accumulation of hyperphosphorylated Tau (pTau) (Figure 1h,l,m and Figure S2c, Supporting Information), and significant reduction in neuronal numbers (Figure 1i).^[^ ^4b,c^ ^]^ Interestingly, we also observed a discernable colocalization between microglia and pTau and further elevated tauopathy, synaptic impairment, and neurodegeneration upon proinflammatory microglial engagement in both AD minibrains (Figure 1c) and AD tissues (Figure 1n and Figure S2d, Supporting Information).

### 2.2 H_2_O_2_ and IFNγ in AD Minibrains Promoting Neurodegenerative Phenotype Transition in Microglia

**Figure 2:**
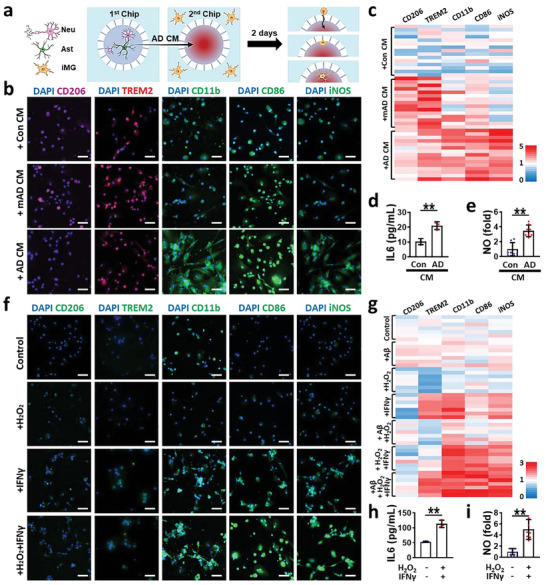
Combined effects of H_2_O_2_ and IFNγ on the transition into proinflammatory microglia in human AD minibrains. a) Schematic illustration representing the induction of microgliosis by using condition medium from AD minibrains (AD CM). Microglia were recruited and activated within 2 d in response to AD CM added to the central chamber. b) Immunofluorescent images showing the transition of homeostatic microglia into beneficial phenotypes (CD206, TREM2) under a conditioned medium of mild human AD minibrain (+ mAD CM) or detrimental phenotype (CD11b, CD86, iNOS) under a conditioned medium of human AD mini‐brain (+AD CM). c) Heatmap presenting iMG phenotype transition matching to AD severity along with AD progression. d) IL6 (two‐tailed unpaired *t*‐test, *n* = 3) and e) NO (two‐tailed unpaired *t*‐test, *n* = 8) augmented by iMGs under the severe AD mini‐brains. f) Immunofluorescent images showing the individual or combined effects of critical soluble factors, such as IFNγ (10 ng mL^−1^) and H_2_O_2_ (10 × 10^−6^ m), selected from AD CM contributing to the neurodegenerative transition. We also added soluble Aβ40 (100 ng mL^−1^) and Aβ42 (10 pg mL^−1^), the major components found in both mAD and AD mini‐brains. g) Heatmap representing the contribution of IFNγ to neurodegenerative phenotype transition. h) IL6 (two‐tailed unpaired *t*‐test, *n* = 3) and i) NO (two‐tailed unpaired *t*‐test, *n* = 8) released by iMGs exposed to IFNγ and H_2_O_2_. Scale bars, 50 µm. All data represents means ± SD. *, *P* < 0.05; **, *P* < 0.01.

Although neuroinflammation is closely correlated with AD progression from mild to severe stages,^[^ ^6a,b^ ^]^ the specific functions of microglia at each of these clinical stages remains unclear. In order to model and clarify the stage‐specific responses of microglia to AD pathology, we mimicked microenvironments of healthy, early‐stage AD, and late‐stage AD human brains by treating iMGs with conditioned medium from human minibrains: control mini‐brains (Con CM); mild AD mini‐brains matured for 6 weeks (mAD CM); and AD minibrains matured for 9 weeks (AD CM), respectively (**Figure** **2**a–c). We regarded AD cultures at 6 weeks to mimic early‐stages of mild AD based on mild pathological signatures of soluble Aβ40 (26 ng mL^−1^), Aβ42 (4.3 pg mL^−1^), and mild oxidative stress (H_2_O_2_, under ≈1× 10^−6^ m) without notable neuronal death. AD mini‐brains at 9 weeks were regarded to model late‐stages of AD based on more severe AD signatures of oxidative stress (H_2_O_2_, 5.9 × 10^−6^ m), proinflammatory mediator (IFNγ, 6.2 ng mL^−1^), the phosphate tau aggregation, and discernable neuronal death.^[^ ^19^, ^20^ ^]^ Afterward, we investigated microglial morphogenesis and phenotype markers for anti‐inflammatory microglia (cluster of differentiation 206 (CD206)),^[^ ^19^, ^21^ ^]^ disease‐associated microglia (triggering receptor expressed on myeloid cells 2 (TREM2)),^[^ ^9^, ^10^ ^]^ and proinflammatory microglia (cluster of differentiation 11b (CD11b) and CD86) (Figure 2b).^[^ ^19^, ^21^, ^22^ ^]^ The heatmap quantification of immunocytochemical imaging showed the microglial transition from homeostatic to reactive status based on the marker changes: resting status of iMGs under “+Con CM,” neuroprotective status under “+mAD CM” (CD206^high^TREM2^high^CD11b^low^CD86^low^), and neurodegenerative status under “+AD CM” (CD206^low^TREM2^med^CD11b^high^CD86^high^) (Figure 2c). Correspondingly, proinflammatory response markers were notably found in iMGs under “+AD CM” such as inducible nitric oxide synthase (iNOS) (Figure 2b,c), interleukin‐6 (IL6) (Figure 2d), and nitric oxide (NO) (Figure 2e), each of which has been shown to induce neurotoxicity.^[^ ^21^, ^23^ ^]^

To further elucidate the underlying mechanism of neuroprotective to neurodegenerative microglia transition, we investigated chemokines (Figure S3c, Supporting Information) and cytokines (Figure S3d, Supporting Information) in AD CM. In the AD models, reactive astrocytes (GFAP‐positive) produced H_2_O_2_ as well as chemokines (CCL1 and CCL2), which can activate and recruit microglia toward the AD models.^[^ ^4b,c^ ^]^ In addition, the reactive astrocytes and neurons in severe AD models released significant levels of proinflammatory cytokines, particularly interleukin‐1 beta (IL1β) (2.8‐fold), IL6 (4.7‐fold), tumor necrosis factor‐alpha (TNFα) (5.8‐fold), and IFNγ (6.3‐fold), which likely further contribute to the neurodegenerative polarization of microglia.^[^ ^4^, ^19^ ^]^ Next, we investigated the correlation of the microglial phenotype transition with above mentioned soluble factors (Figure 2f,g and Figure S5, Supporting Information) and selected H_2_O_2_ (10 × 10^−6^ m) and IFNγ (10 ng mL^−1^) as the major contributors. We also added soluble Aβ40 (100 ng mL^−1^) and Aβ42 (10 pg mL^−1^), the major component found from the early to late AD stage. We selected the concentration ranges measured in AD mini‐brains. We plotted the immunostaining results (Figure 2f) and the heatmap data (Figure 2g) in the order from neuroprotective (CD206, TREM2) to neurodegenerative (CD11b, CD86, iNOS) phenotype markers. The single treatment of Aβ40+42 or IFNγ resulted in the promotion of neuroprotective markers. Given the fact that IFNγ was not detected while Aβ was dominant in the mAD models, Aβ would be the major source to induce neuroprotective microglia in the early‐to‐moderate stages. Furthermore, there were synergistic effects of H_2_O_2_ and IFNγ on the neurodegenerative phenotype transition, increasing the expression of CD11b, CD86, iNOS (Figure 2f,g) and the production of IL6 and NO (Figure 2h,i).

### 2.3 IFNγ‐Induced Nrf2 Downregulation in Microglia during the Neurodegenerative Transition

**Figure 3:**
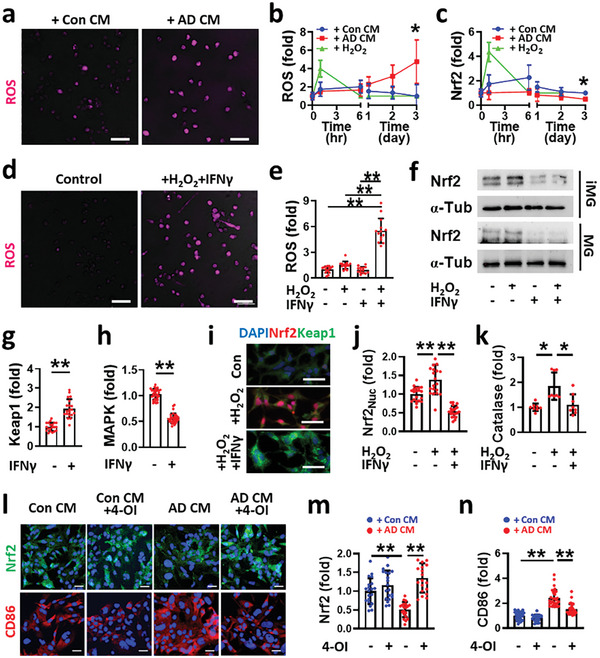
IFNγ downregulates microglial Nrf2 via Keap1 upregulation in human AD minibrains. ROS accumulation within iMG cells under either a,b) a severe human AD mini‐brain (+AD CM) (two‐tailed unpaired *t*‐test, *n* = 16) or d,e) a combined treatment of IFNγ (10 ng mL^−1^) and H_2_O_2_ (10 × 10^−6^ m) (one‐way ANOVA with Tukey's multiple‐comparisons test, *n* = 15). c,f) The reduced levels of Nrf2 in iMG treated by either c) AD CM (two‐tailed unpaired *t*‐test, *n* = 8) or f) IFNγ + H_2_O_2_. IFNγ increased g) Keap1 (two‐tailed unpaired *t*‐test, *n* = 20) while decreased h) MAPK levels in MG (two‐tailed unpaired *t*‐test, n = 30). i) Fluorescent images and j) quantitative analysis confirm promotion of Keap1 while inhibiting Nrf2 from nucleus internalization by IFNγ (one‐way ANOVA with Tukey's multiple‐comparisons test, n = 20). k) Expression level of Catalase in MG was decreased by IFNγ (one‐way ANOVA with Tukey's multiple‐comparisons test, *n* = 8). Inhibition of Keap1 by 4‐OI recovered Nrf2 level and reduced neurodegenerative phenotype transition under AD CM. l) Fluorescent images and quantitative results for m) Nrf2 (one‐way ANOVA with Tukey's multiple‐comparisons test, *n* = 20) and n) CD86 (one‐way ANOVA with Tukey's multiple‐comparisons test, *n* = 30). Scale bars, a) 50 µm, d) 50 µm, i) 20 µm, l) 50 µm. All data represents means ± SD. *, *P* < 0.05; **, *P* < 0.01.

Microglia clear amyloid plaques and tau aggregates producing reactive oxidative species (ROS),^[^ ^24^ ^]^ which can be removed by intracellular antioxidation mechanism in normal conditions. Therefore, the dysregulation in the antioxidation, can result in the accumulation of intracellular oxidative stress and trigger the nuclear factor kappa B (NFκB)‐axis, leading to neurodegenerative phenotype polarization.^[^ ^4^, ^25^ ^]^ Correspondingly, the level of intracellular ROS was significantly elevated in iMG under AD CM compared to under Con CM (**Figure** **3**a,b). To understand the correlation of the ROS with to neurodegenerative phenotype polarization, we first assessed the level of nuclear factor erythroid‐2‐related factor 2 (Nrf2), a central transcriptional factor controlling multiple antioxidative enzymes.^[^ ^26^ ^]^ To this end, iMGs were treated with either AD CM or Con CM while monitoring the level of Nrf2 in iMGs. We treated iMGs with H_2_O_2_ (10 × 10^−6^ m, the concentration in the AD CM) as a positive control inducing ROS. Our data showed that the treatment of Con CM or H_2_O_2_ maintained the basal level of ROS (Figure 3a,b) and transiently activated Nrf2 (Figure 3c) in iMGs as normal. However, Nrf2 was not activated but further decreased in iMGs under AD CM, which would contribute to ROS accumulation in iMGs. Furthermore, the combined treatment of IFNγ and H_2_O_2_, the AD condition promoting the neurodegenerative transition mostly, dramatically induced the ROS accumulation in iMGs compared to the single treatment of H_2_O_2_ (Figure 3d,e) as the addition of IFNγ decreased Nrf2 expression in iMG (Figure 3f). We next investigated the underlying mechanisms of Nrf2 downregulation driven by IFNγ. Interestingly, the addition of IFNγ increased Kelch‐like ECH‐associated Protein 1 (Keap1) expression, a major repressor binding to Nrf2,^[^ ^27^ ^]^ in MG (Figure 3g). We also found that IFNγ downregulated mitogen‐activated protein kinases (MAPK) (Figure 3h), another key modulator phosphorylating Nrf2 and dissociating Nrf2 from Keap1.^[^ ^28^ ^]^ It should be noted that H_2_O_2_ did not affect the MAPK level in the microglia (Figure S6, Supporting Information). We presumed that the promotion of Keap1‐Nrf2 interaction driven by IFNγ contributed to the prevention of Nrf2 translocalization into nucleus (Figure 3i,j) and the reduction of antioxidant as the Catalase (Figure 3k) even in the presence of oxidative stress (H_2_O_2_), as reported previously.^[^ ^29^ ^]^ Correspondingly, the treatment of Four‐octyl itaconate (4‐OI), a Keap1 alkylator promoting the release of Nrf2 from Keap1, increased the level of Nrf2 (Figure 3l,m) while decreased the level of CD86 (Figure 3l,n).

**Figure 4:**
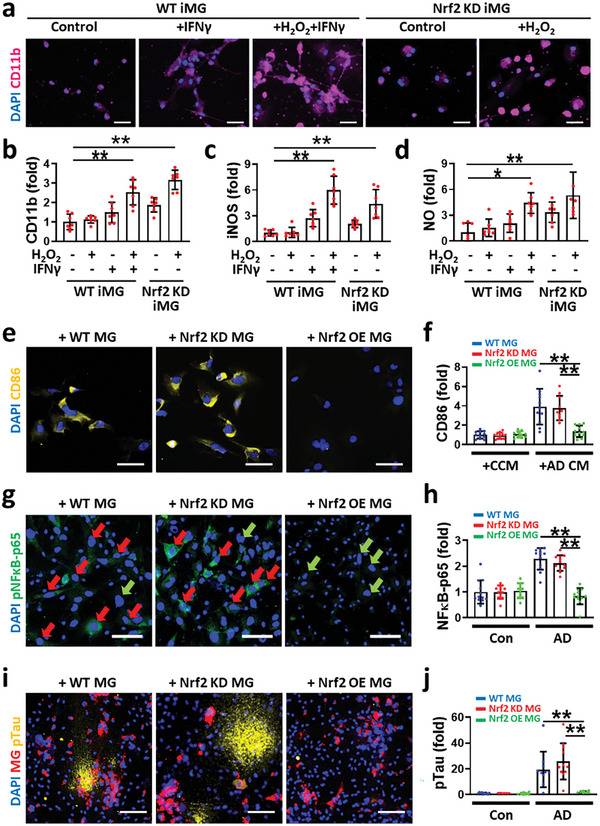
Nrf2 critically involves in the transition of microglial phenotype in AD mini‐brains. a,b) Induction of neurodegenerative phenotype (CD11b^high^) in Nrf2‐knockdown microglia (Nrf2 KD iMG) treated with H_2_O_2_ (10 × 10^−6^ m), which was comparable to control microglia (WT iMG) treated with both IFNγ (10 ng mL^−1^) and H_2_O_2_ (10 µM) (one‐way ANOVA with Tukey's multiple‐comparisons test, *n* = 7). c,d) The levels of the iNOS expression and NO production assessed by fluorescent staining were plotted in (c,d) (one‐way ANOVA with Tukey's multiple‐comparisons test, *n* = 7), respectively. e,f) Neurodegenerative transition of wild type microglia (WT MG) and Nrf2‐downregulated microglia (Nrf2 KD MG) while no transition of Nrf2‐overexpressed microglia (Nrf2 OE MG) by treatment of AD CM (one‐way ANOVA with Tukey's multiple‐comparisons test, *n* = 11). g,h) Activation of the classical NFκB pathway in AD models with WT MG and Nrf2 KD MG, which was rescued by the addition of Nrf2 OE MG (one‐way ANOVA with Tukey's multiple‐comparisons test, *n* = 12). We marked NFκB‐activated microglia with red and inactivated microglia with green. i,j) Promotion or inhibition of tauopathy driven by Nrf2 KD MG or Nrf2 OE MG (one‐way ANOVA with Tukey's multiple‐comparisons test, n = 10). All data represents means ± SD. *, *P*<0.05; **, *P*<0.01. Scale bars: a) 50 µm, e) 100 µm, g) 100 µm, i) 100 µm.

Recent studies have proved that Nrf2 inhibition induced inflammatory microglia serving neurodegenerative roles.^[^ ^30^ ^]^ At this point, we set a hypothesis that IFNγ may impair the Nrf2 activation via Nrf2‐Keap1 axis, failing to remove intracellular oxidative stressors and contributing to the microglial neurodegenerative phenotype transition. To test this hypothesis, we prepared Nrf2‐downregulated iMGs (Nrf2 KD iMG), transiently transfected by Nrf2‐specific small interfering RNA (siNrf2) (Figure S7, Supporting Information), and investigated whether Nrf2 KD iMGs were transited into neurodegenerative phenotype under the microenvironment with oxidative stress. For the control (WT iMG), we treated iMGs with nontargeting scrambled siRNA (siCon). In accordance with the induction of neurodegenerative microglia in WT iMGs treated with IFNγ+H_2_O_2_, we observed the polarization of neurodegenerative phenotype in Nrf2 KD iMGs treated with H_2_O_2_ (**Figure** **4**a,b). Moreover, both WT iMG+IFNγ+H_2_O_2_ and Nrf2 KD iMG+H_2_O_2_ induced expression of iNOS (Figure 4c) and elevated production of NO by iMGs (Figure 4d). We next investigated how the Nrf2 downregulation in microglia could affect the neurodegenerative phenotype transition in AD mini‐brains. To this end, we first prepared Nrf2‐downregalated microglia (Nrf2 KD MG) or Nrf2‐overexpressed microglia (Nrf2 OE MG) by treating MGs with siNrf2 constructs or lentiviral vector with Nrf2‐overexpressing gene (NM_010902). Afterward, we seeded them to Control or AD mini‐brains and monitored the expression level of neurodegenerative phenotype marker (CD86). Our data revealed that both wild type microglia (WT MG) and Nrf2 KD MG treated with AD CM expressed the increased level of CD86 (Figure 4e,f). In addition, Nrf2 overexpression significantly reduced the neurodegenerative microglia under AD CM‐treated conditions compared to WT MG and Nrf2 KD MG; yet could not revert them to the neuroprotective phenotype (Figure S8, Supporting Information). Our data confirmed that downregulation of Nrf2 could contribute to the neurodegenerative transition under AD‐like conditions. We also checked any changes in the levels of phosphorylated NFκB‐p65 in the microglia, the active form of NFκB known to promote the neurodegenerative phenotype (Figure 4g,h).^[^ ^31^ ^]^ Our data showed that the classical NFκB pathway was activated in the microglia expressing low level of Nrf2 such as MG and Nrf2 KD MG. Correspondingly, Nrf2 KD MG slightly increased the pTau accumulation compared to WT MG while Nrf2 OE MG significantly blocked the the pTau accumulation in AD mini‐brains (Figure 4i,j). We further tested whether the treatment of H_2_O_2_ scavenger (AAD), as an antioxidant,^[^ ^4c^ ^]^ could decrease the glial reactivity as well as neuronal death in AD minibrains (Figures S9 and S10, Supporting Information). It should be noted that the treatment of AAD could remove H_2_O_2_ completely (Figure S9a, Supporting Information) and reduce IFNγ level significantly (Figure S9b, Supporting Information). Our data revealed that the treatment of AAD significantly decreased reactive astrocytes (Figure S10a,b, Supporting Information) and neurodegenerative microglia (Figure S10a,c, Supporting Information). Correspondingly, we found that the treatment of AAD prevented pTau accumulation in AD minibrains (Figure S10d, Supporting Information) and rescued neurons in AD mini‐brains (Figure S10e, Supporting Information). Collectively, we concluded that IFNγ‐mediated Nrf2 downregulation promoted ROS accumulation, proinflammatory phenotype transition in human microglia through the classical NFκB pathway, and neurodegeneration in AD mini‐brains. In addition, our data validated the use of antioxidant targeting IFNγ‐Nrf2 axis as a promising therapeutic strategy for AD treatment.

### 2.4 Demonstration of Neurodegenerative Microglia Showing Quiescent Nrf2 Activity in Brain Samples from AD Patients and 5XFAD Mice

**Figure 5:**
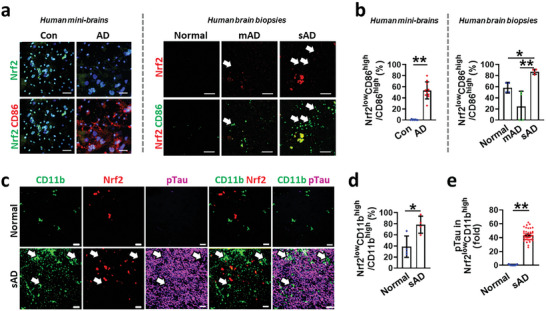
Downregulation of Nrf2 in proinflammatory microglia correlating with pTau accumulation in brain tissues of AD patients. a) Immunofluorescent images and b) quantitative analysis showing the presence of Nrf2^low^CD86^high^ microglia in AD minibrains (two‐tailed unpaired *t*‐test, *n* = 10) and human brain biopsies (one‐way ANOVA with Tukey's multiple‐comparisons test, *n* = 4). Nrf2^low^CD86^high^ microglia were mGarked with white arrows. c) Immunofluorescent images of human brain tissues showing the increase of Nrf2^low^CD11b^high^ microglia (white arrows) near pTau accumulated regions in sAD. d) Quantitative analysis showing the significant increase of Nrf2^low^CD11b^high^ among the total CD11b^high^ microglia (two‐tailed unpaired *t*‐test, *n* = 4) near pTau accumulations in sAD compared to normal. e) Quantification of pTau level near Nrf2^low^CD11b^high^ microglia in sAD brain samples (two‐tailed unpaired *t*‐test, *n* = 40). Scale bars, 20 µm. All data represents means ± SD. *, *P*<0.05; **, *P*<0.01.

In this study, we revealed that IFNγ‐driven Nrf2 downregulation was the key contributor activating neurodegenerative microglia in H_2_O_2_‐enriched AD mini‐brains. We next investigated the induction of any neurodegenerative microglia with the Nrf2 impairment in AD minibrains and brain tissues of AD patients (**Figure** **5**a,b). We categorized AD patient samples into the mild AD (mAD) and the severe AD model (sAD) according to the severity levels described by the National Institute of Aging Reagan criteria. We validated that sAD expressed the increased level of IFNγ in Figure 1j. We observed that Nrf2^high^CD86^high^ microglia were rarely found but the majority was the Nrf2^low^CD86^high^ microglia (marked with arrows) in both AD mini‐brains and sAD models. Our quantification analysis showed that the population of Nrf2^low^CD86^high^ microglia was reduced in mAD while significantly increased in sAD (Figure 5b). We further validated the presence of Nrf2^low^CD11b^high^ microglia near pTau deposition (Figure 5c) in AD samples. We found that the level of pTau, co‐localized with Nrf2^low^CD11b^high^, was discernably increased in sAD (Figure 5e). Likewise, the Nrf2^low^CD86^high^ microglia were found in 5XFAD mice (Figure S11e,f, Supporting Information), which exhibited a significantly increased level of IFNγ (Figure S11b, Supporting Information) while a decreased level of Nrf2 (Figure S11c,d, Supporting Information) compared to wild type (Normal). To further investigate whether Nrf2 overexpression in microglia could ameliorate AD pathology in animal models, we developed 5XFAD + Nrf2 OE mice, which promoted the Nrf2 level specifically within the microglia. Correspondingly, we observed a significant enhancement in fear conditioning memory in 5XFAD + Nrf2 OE mice, indicating that microglia‐specific overexpression of Nrf2 can positively improve the memory functions in 5XFAD mice (Figure S11g, Supporting Information). Taken together, our study validated the impaired Nrf2 activation in proinflammatory microglia and correlation with neurodegeneration in both brain samples from AD patients and 5xFAD mice.

## 3 Discussion

In this study, we developed pathophysiologically relevant and easily‐accessible models of human AD minibrains reconstructing progressive AD signatures (Figure 1a–i) in comparison with human AD patients (Figure 1j–n) and AD mouse models (Figures S2 and S11, Supporting Information). We created 3D human AD minibrains by try‐culturing human neuroprogenitor‐derived neurons, astrocytes, and hiPSC‐driven microglia in a microfluidic platform. As our human AD models were being matured, they progressively showed AD signatures like in early to late AD stages. In addition, our microglial chemotactic platform enabled separating the subset of microglia in response to AD models. Our data validated that more than 97.6%, 95.8%, and 94.0% of recruited microglia in response to AD CM in the central chamber expressed the higher level of CD11b, CD86, and iNOS, respectively (Figure 2b). From the purified microglia, we could correlate microglial phenotype changes with AD progression: neuroprotection in the early‐staged AD while neurodegeneration with the late‐staged AD. We observed synaptic impairment, hyperphosphorylated‐tau accumulation, and neuronal loss as the evidence of neurodegeneration.

Our study revealed the combined effects of IFNγ and H_2_O_2_ derived from co‐cultured AD neurons and astrocytes, which promoted the accumulation of ROS followed by the transition into proinflammatory phenotype in the late‐staged AD mini‐brains. There are contradictory results regarding the roles of IFNγ in the pathogenesis of neurological disorders. Several studies showed that IFNγ can serve neuroprotective roles as antiviral and antitumor activities.^[^ ^32^ ^]^ IFNγ can also maintain homeostasis in the brains providing neuroprotection such as growth, maturation, and differentiation of neuronal cells and innate immune cells including microglia.^[^ ^33^ ^]^ On the other hand, IFNγ can promote proinflammation and neuronal degeneration. For instance, IFNγ is known to promote NADPH oxidase 1 (NOX1) or NADPH oxidase2 (NOX2)‐mediated oxidative stress in microglia as well as activate signal transducer and activator of transcription 1 (STAT1) and NFκB followed by iNOS signaling pathways.^[^ ^34^ ^]^ In this study, we for the first time revealed that IFNγ promoted the accumulation of oxidative stress in microglia under AD conditions by Keap1‐mediated Nrf2 downregulation (Figure 3g–j) leading to the reduction of antioxidant enzyme as the Catalase (Figure 3k). We previously proved that the removal of IFNγ from AD CM by using IFNγ‐neutralizing antibody prevented microglial proinflammation and consequently improved the neural viability, which was clear enough in our in vitro model.^[^ ^4b^ ^]^ We also revealed the microglial phenotype transition leading to neurodegeneration via Keap1‐Nrf2 axis by using Nrf2 KD MG or Nrf2 OE MG. To further validate the direct effects of IFNγ contributing to microglial phenotype transition, further studies with IFNγ receptor (IFNGR) knock‐out or knock‐down in microglia would be required.

We next performed co‐validation of quiescent Nrf2 activity of neurotoxic microglia in IFNγ‐enriched AD mini‐brains, AD patients (Figure 5), as well as 5XFAD mice (Figure S11, Supporting Information). We found that the population for Nrf2^low^CD86^high^ microglia was significantly increased in AD mini‐brains and AD patients (Figure 5b). Compared to human AD mini‐brains (≈55%) and biopsies (≈80%), the population for Nrf2^low^CD86^high^ microglia was lower in 5XFAD mice (Figure S11f, Supporting Information, ≈30%). We attributed the modest activation of proinflammatory microglia in 5XFAD mice to the different genetic background from human. In many cases of experimental AD mouse models forming Aβ plaques, the formation of neurofibrillary tangles (NFTs) in neurons was not reproduced well (Figures S2 and S11, Supporting Information).^[^ ^35^ ^]^ In addition, significant neuronal loss was observed in the limited area of AD mice while severe loss in the entire brain was frequently found in human AD patients.^[^ ^17b^ ^]^ In this matter, we believe our humanized AD mini‐brain, sharing the genetic materials of human, can reproduce AD pathology and offer a reliable and effective AD model system.

## 4 Conclusion

In summary, we identified key regulators in the microglial phenotype transition by reconstituting AD models and monitoring multiple human microglial markers for the first time. Soluble amyloid‐beta induced microglia to retain neuroprotective phenotypes including phagocytic activity at early‐to‐moderate stages. The synergistic effect of IFNγ and H_2_O_2_ was effective in microglia neurodegenerative phenotype transition while producing neurotoxic NO and proinflammatory cytokines at the severe AD stage. IFNγ‐mediated Keap1 activation followed by Nrf2 downregulation induced the accumulation of oxidative stress in microglia and triggered the transition into neurodegenerative phenotype. Importantly, we further confirmed the impairment of Nrf2 activation in neurodegenerative microglia in AD human patients and 5XFAD mice as well.

## 5 Experimental Section

### Chip Fabrication

The chemotactic chip was employed to create AD brain models. Details of the chip design were described in the previous study.^[^ ^36^ ^]^ To fabricate a mold of the devices, a SU‐8 negative photoresist (MicroChem, Round Rock, TX), was sequentially patterned using photolithography on a silicon wafer. A mixture of base and curing agent of Sylgard 184 A/B polydimethyl‐siloxane (PDMS) (Dow Corning, Midland, MI) was poured onto the SU‐8 mold to replicate the microstructures. The cured PDMS replica was removed from the mold, and holes were created for fluid reservoirs. Plastic chambers for medium reservoirs were fabricated with a computer‐controlled Zing laser cutter (Epilog Laser, Golden, CO) with a 6 mm thick acrylic plate. The replicated PDMS and plastic layers were glued together using PDMS. The resultant assembly was irreversibly bonded to a customized glass‐bottomed uni‐well plate (MatTek, Ashland, MA) by oxygen plasma treatment (Plasma Etch, Carson City, NV). In prior to the cell culture on the device, each chamber was coated with 1% (v/v) Matrigel matrix (Dow Corning) diluted in DMEM/F‐12 (Life Technologies, Grand Island, NY) for 1 h and washed it with Dulbecco's phosphate‐buffered saline (DPBS, Lonza, Hopkinton, MA) thoroughly.

### Cell Culture

ReN cell VM human neural progenitor cells (hNPCs, EMD Millipore, Billerica, MA) were plated onto the culture flask coated with 1% (v/v) Matrigel in ReN cell culture medium, DMEM/F12 supplemented with 2 mg heparin (StemCell Technologies, Vancouver, BC, Canada), 2% (v/v) B27 neural supplement (Life Technologies, Grand Island, NY), 20 mg EGF (Sigma‐Aldrich, St Louis, MO), 20 mg bFGF (Stemgent, Cambridge, MA), and 1% (v/v) penicillin/streptomycin/amphotericin‐B solution (Lonza), and incubated at 37 °C supplied with 5% CO_2_. Cell culture medium was changed every 3 d until cells were confluent. To harvest hNPCs, the cells were washed with DPBS, detached using Accutase (Life Technologies), and resuspended in fresh culture medium. SV40 human adult microglia cells (MG, Applied Biological Materials Inc. (ABM), Richmond, BC, Canada) were cultured in MG media, PriGrow III (ABM) supplemented with 10% (v/v) fetal bovine serum (FBS, Thermo Fisher Scientific, Waltham, MA), and incubated at 37 °C supplied with 5% CO_2_. Cell culture medium was changed every 2 d until cells were confluent. To harvest MGs, the cells were washed with DPBS, detached using 2.5% Trypsin (Thermo Fisher Scientific), and resuspended in fresh culture medium.

### Preparation of Human Neural Progenitor Cells Expressing APPSL

ReN hNPCs were cultured on six‐well plate and transduced with commercially available APPSL‐GFP Alzheimer's lentiviruses (EMD Millipore) to develop ReN cells producing high levels of Aβ (AD hNPCs) through overexpression of a variant of the human amyloid precursor protein (APP) containing K670N/M671L (Swedish) and V717I (London) FAD mutations (APPSL). Briefly, ReN cells were transduced with 5 µL of viral solution containing APPSL‐GFP lentivirus (1 × 10^9^ IFU mL^−1^) and polybrene (EMD Millipore, 2 mg mL^−1^) and incubated for 24 h. Expression of infected genes was confirmed by fluorescence. For the control counterpart, ReN cells were transduced with the control GFP construct (LentiBrite GFP Control, EMD Millipore) to develop control hNPCs. Afterward, cells were washed with PBS three times and performed a second virus infection by adding the viral solution in the fresh medium. After 24 h, cells were washed with PBS three times and added fresh medium. After 2 d of incubation, the transgene positive cells were enriched by FACS sorting (BD FACS Aria II, BD Biosciences).

### Preparation of Human iPSC‐Microglia (iMGs)

iPSC‐microglia were generated as previously described.^[^ ^10^, ^14^ ^]^ Briefly, iPSCs were differentiated into hematopoietic progenitors (iHPCs) using the STEMdiff Hematopoesis kit (StemCell Technologies). After 11 d in culture, CD43^+^ iHPCs were transferred into a microglia differentiation medium containing DMEM/F12, 2× insulin‐transferrin‐selenite, 2× B27, 0.5× N2, 1× Glutamax, 1× non‐essential amino acids, 400 × 10^−6^ m monothioglycerol, and 5 µg mL^−1^ human insulin. Medium was added to cultures every other day and supplemented with 100 ng mL^−1^ IL‐34, 50 ng mL^−1^ TGF‐β1, and 25 ng mL^−1^ M‐CSF (Peprotech, Cranbury, NJ) for 28 d. In the final 3 d of differentiation 100 ng mL^−1^ CD200 (Novoprotein, Summit, NJ) and 100 ng mL^−1^ CX3CL1 (Peprotech) were added to culture to further mature iMGs.

### Preparation of Nrf2‐Modulated Microglia

Cells were cultured on six‐well plate. To knockdown Nrf2 gene expression, iMGs (or MGs) were treated with 10 × 10^−6^ m of Nrf2‐specific small interfering RNA (siNrf2, Origene, Rockvile, MD) for 8 h. For the control, cells were treated with 10 × 10^−6^ m of nontargeting scrambled siRNA (siCon, Origene). The silencing of Nrf2 in iMGs was confirmed by immunostaining (Figure S7a, Supporting Information) and western blotting analysis (Figure S7b, Supporting Information) after 48 h of trasfection. To prepare Nrf2‐overexpressing microglia (Nrf2 OE MG), MGs were transduced with 50 µL of viral solution containing Nrf2‐GFP lentivirus (1 × 10^8^ IFU mL^−1^) and polybrene (EMD Millipore, 2 mg mL^−1^) and incubated for 24 h. For the control counterpart, MGs were transduced with 50 µL of viral solution containing GFP lentivirus (1 × 10^8^ IFU mL^−1^) and polybrene (EMD Millipore, 2 mg mL^−1^). Afterward, cells were washed with PBS three times and performed a second virus infection by adding the viral solution in the fresh medium. After 24 h, cells were washed with PBS three times and added fresh medium. After 2 d of incubation, the transgene positive cells were enriched by FACS sorting (BD FACS Aria II).

### Preparation of Human AD Brain Models

The 3D human AD model was utilized to investigate the adverse microgliosis on human AD brains. AD hNPCs were plated in the differentiation medium at the density of 1× 10^7^ cells mL^−1^. Next, the cell solution was mixed with Matrigel in 1:5 ratio (v/v); and 10 µL of the mixture was added to the central chamber of the microfluidic device to achieve 3D cultured ReN cells in 20% Matrigel. For the control counterpart, control hNPCs were added to the central chamber. Additional 100 µL of ReN cell culture medium without EGF and bFGF (differentiation medium) was added to both the central chamber and two annular chambers per a device. The microfluidic devices were placed in a 5% CO_2_ cell culture incubator at 37 °C. One‐half volume of the differentiation medium in the central chamber was replaced every 3.5 d until the progenitor cells were fully differentiated into neurons and astrocytes (approximately 2 weeks). The replacement of one half of medium every 3.5 d was designed to prevent any unexpected cell damage, caused by the shortage of nutrients in the 3D cultured models. At the week 9 (W9), entire medium was replaced to the iMG maintenance medium (iCell Microglia Complete Maintenance medium, Fujifilm, Japan) or MG medium supplemented with 2% (v/v) FBS, and iMG or MG were added to the annular chamber at the cell seeding density of 5000 cells per device, respectively. It should be noted that 2% (v/v) FBS could maintain the viability of MG,^[^ ^4b^ ^]^ but not induce migration between chambers (Figure S12, Supporting Information). To avoid unexpected chemotaxis caused by medium gradients, the central chamber medium was also replaced to the same medium added to the annular chamber. Prior to proceeding experiments, it was confirmed that there was no migration in control groups. The microfluidic devices were incubated in a 5% CO_2_ cell culture incubator at 37 °C for 2 d to complete microglia migration in response to the soluble factors from the central chamber. For the single‐cultured microglia models, soluble factors were added to the central chamber and incubated them in a 5% CO_2_ cell culture incubator at 37 °C for 2 d. The number of recruited microglia was monitored in the central chamber for 2 d under the fully automated fluorescence microscope (Nikon TiE microscope, Nikon, Melville, NY). It was observed that the increased number of iMGs and MG were activated and migrated to the central chamber of AD mini‐brains in 2 d compared to control counterpart (Figure 1b).

### Multicytokine Assay

Upon the completion of microglia migration to the central chamber, 1 mL of each conditioned medium of control or AD models was collected and employed at the end point of experiment. The instructions were followed provided by the manufacturer to assess chemokines and cytokines released by control and AD models by using an ARY005B human cytokine array kit (R&D systems, Minneapolis, MN). The chemiluminescence signals were detected by using a ChemiDocTM Imaging System (Bio‐Rad, Hercules, CA).

### Enzyme‐Linked Immunosorbent Assay (ELISA)

100 µL of each conditioned medium of control or AD models was collected and employed at the end point of experiment. The instructions were followed provided by the manufacturer to assess the levels of Aβ40, Aβ42, IFNγ, and IL6 in the conditioned medium, by using Human Aβ40 ELISA kit (Invitrogen, Waltham, MA), Human Aβ42 ELISA kit (Invitrogen), Human IFNγ ELISA kit (Invitrogen), and Human IL6 ELISA kit (Invitrogen), respectively. The absorbances at 450 nm (primary signals) and 650 nm (reference signals) were assessed using a microplate reader (Synergy HT, BioTek Instruments, Winooski, VT).

### Reactive Oxygen Species (ROS) Measurement

iMGs were treated with 9 weeks AD conditioned medium or IFNγ and/or H_2_O_2_ for 2 d and washed cells with PBS for three times. Afterward, the microglia was stained with 5 × 10^−6^ m CellROX (Thermo Fisher Scientific) for 20 min at 37 °C, fluorescent probes detecting the intracellular ROS. Cells were washed with PBS for three times and measured fluorescent intensity by using a fluorescence microscope (Nikon TiE microscope, Nikon) equipped with a TRITC filter. Any fold changes were investigated in the fluorescent intensity representing the microglial ROS by using a NIS‐Elements software.

### Nitric Oxide (NO) Measurement

Fold changes in NO concentration in microglia were monitored using the fluorescent NO probe, difluorofluorescein‐FM diacetate (DAF‐FM DA, Thermo Fisher Scientific). Briefly, cells were washed with PBS and treated with 5 × 10^−6^ m DAF‐FM DA diluted in the differentiation medium supplemented with 5% (v/v) FBS for 30 min at 37 °C. Microglia with PBS was rinsed three times to remove excessive probes, added the fresh differentiation medium supplemented with 5% (v/v) FBS, and incubated them for an additional 30 min to complete the de‐esterification of diacetates in the cells. Afterward, NO was detected by using a fluorescence microscope (Nikon TiE microscope, Nikon) equipped with a FITC filter. The fluorescent intensity was analyzed representing NO by using a NIS‐Elements software.

### Immunocytochemistry

For immunostaining of 3D cultured cells in the device, the models were rinsed with PBS twice and fixed with 4% paraformaldehyde (PFA, Electron Microscopy Sciences, Hatfield, PA) for 30 min at RT. Cells were then rinsed with PBS two times with 10 min intervals and incubated in the permeabilizing solution, PBS solution supplemented with 0.1% (v/v) Triton X‐100 and 0.1% (v/v) Tween 20 (PBSTT), for 30 min at RT. Cells were next washed with PBS three times with 10 min intervals and incubated in the blocking solution, PBS solution supplemented with 0.1% (v/v) Tween 20 and 3% (v/v) human serum albumin (BSA), for 2 h at RT. Cells were again washed with PBS three times with 10 min intervals and incubated with the primary antibody diluted in the blocking solution. Details of primary and secondary antibodies in terms of dilution ratio and other information were summarized in Table S2 (Supporting Information). After secondary antibody reaction, the devices were washed seven times with PBS supplemented with 0.1% (v/v) Tween 20 (PBST) with 10 min intervals and examined under a fluorescence microscope (Nikon TiE microscope, Nikon). The intensity of immunoreactivity was analyzed by using a NIS‐Elements software.

### In Vitro Toxicology Assay

To assess any cytotoxicity driven by neuroinflammation, lactic dehydrogenase (LDH)‐based toxicology assay was employed. Briefly, 100 µL of each conditioned medium of control or AD models was collected at the end point of experiment, and then mixed the conditioned medium with 100 µL of LDH assay reaction mixture (Sigma‐Aldrich). After 1 h incubation at room temperature, the stop buffer was added to block the enzyme reaction and measured the signals at 490 nm by using a microplate reader (Synergy HT, BioTek Instruments).

### Human Tissues

Normal and AD human brain samples (Table S1, Supporting Information) were from the Boston University Alzheimer's Disease Center (BUADC) and Genome Science Institute (BUGSI). Institutional review board approval for ethical permission was obtained through the BUADC and CTE center. This study was reviewed by the Institutional Review Board of the Boston University School of Medicine (Protocol H‐28974) and was approved for exemption because it only included tissues collected from post‐mortem subjects not classified as human subjects. The study was performed in accordance with institutional regulatory guidelines and principles of human subject protection in the Declaration of Helsinki. Postmortem brain tissues were selected in a coronal plane at 10 µm. The neuropathological diagnosis for AD was performed by board‐certified neuropathologists based on the National Institute of Aging Reagan criteria and included intermediate or high probability. In cases of AD patients, the Braak Stages of III to IV and V to VI were divided to mild cognitive impairment AD (neuropathological and clinical AD: NPCAD) and severe AD cases, respectively.

### mRNA‐Sequencing (RNA‐seq) and Data Analysis

Total RNAs were isolated from the frozen brain tissues using Trizol reagent (Invitrogen). RNA quality was assessed by Agilent 2100 bioanalyzer using the RNA 6000 Nano Chip. Samples were prepared for mRNA‐seq following the Illumina standard protocol. Briefly, 3 µg of total RNA from each sample was used for polyA mRNA selection using streptavidin‐coated magnetic beads, followed by thermal mRNA fragmentation. The fragmented mRNA was reverse‐transcribed to generate cDNA using reverse transcriptase (SuperScript II) and random primers, which was further converted into double‐stranded cDNA. After an end repair process (Klenow fragment, T4 polynucleotide kinase and T4 polymerase), the resulting cDNA was finally ligated to Illumina paired end (PE) adaptors. Using a 2% agarose gel, cDNA libraries ranging in size between 200 and 250 bp were selected, subjected to ten cycles of PCR, and then purified using the QIAquick PCR purification kit (Qiagen, Hilden, Germany). The enriched libraries were diluted with Elution Buffer to a final concentration of 10 × 10^−9^ m. Finally, 8 × 10^−12^ m of the library in each sample was sequenced using the HiSeq2000 with 101 bp sequencing. The RNA‐seq data were analyzed as described previously.^[^ ^37^ ^]^ The expression levels of INFγ and INFγ‐associated genes were presented FPKM (Fragments Per Kilobase of exon per Million) or fold change. The sequencing data from the previous study was deposited to European Nucleotide Archive (ENA) (accession number: PRJEB36676).

### Immunofluorescence and Confocal Microscopy for Human Tissues

Immunofluorescence staining for CD86, Nrf2, and TREM2 was performed on paraffin sections of hippocampus tissues from fixed normal and AD postmortem brains. Briefly, paraffin embedded sections were deparaffinized, rehydrated, and treated with 3% H_2_O_2_ for antigen retrieval. After blocking with TBS‐T contained 5% fetal bovine serum for 1 h, tissue sections were incubated with primary antibodies for 24 h. Details of primary and secondary antibodies in terms of dilution ratio and other information were summarized in Table S2 (Supporting Information). After three times of washing, the slides were incubated with fluorescence‐conjugated secondary antibody. The nuclei were counterstained with DAPI (4′,6‐diamidino‐2‐phenylindole). Images were captured using a Confocal microscope (Nikon A1 microscope, Nikon). The intensity of immunoreactivity was analyzed by using an Image J software (National Institutes of Health).

### Illustrations

All illustrations were generated with a license to Biorender (https://www.biorender.com).

### Statistical Analysis

Data in graphs are presented as mean ± SD unless otherwise stated. The number of samples examined per each group are specified for each set of data in the corresponding figure caption. Statistical significance for comparison of two experimental groups was determined by unpaired two‐tailed *t*‐test using GraphPad Prism 6 (GraphPad Software, La Jolla, CA). The probability value of *p* < 0.05 was considered significant. Statistical significance for multiple comparisons was carried out by One‐way ANOVA with Tukey post‐hoc correction using IBM® SPSS® Statistics Premium 27 (SPSS Inc., Chicago, IL). The probability value of *p* < 0.05 was considered significant. All statistical data were summarized in Table S3 (Supporting Information). The * and **represent *p* > 0.05 and *p* < 0.01, respectively.

### Animal Experiments in Supporting Information

All animal experiments and procedures were approved by the Institutional Animal Care and Use Committee (IACUC) of the Institute for Basic Science (IBS‐2022‐001; Daejeon, Korea) and Sungkyunkwan University (SKKUIACUC2020‐05‐18‐2; Suwon, Korea). All mice were group‐housed in a temperature‐ and humidity‐controlled environment with a 12 h light/dark cycle and had free access to food and water.

## Conflict of Interest

The authors declare no conflict of interest.

## Supporting information

Supporting Information

## Data Availability

The data that support the findings of this study are available from the corresponding author upon reasonable request.
