# Supplementary material for: Neurotoxic Microglial Activation via IFNγ‐Induced Nrf2 Reduction Exacerbating Alzheimer's Disease
Source: Adv Sci (Weinh). 2024 Mar 14;11(20):2304357. doi: 10.1002/advs.202304357 (PMC11132036; doi:10.1002/advs.202304357)
Supplement: Supplementary file 1 — Supporting Information [file ADVS-11-2304357-s001.pdf]

ADVANCED SCIENCE
Open Access

Supporting Information

for *Adv. Sci.*, DOI 10.1002/advs.202304357

Neurotoxic Microglial Activation via IFNγ-Induced Nrf2 Reduction Exacerbating Alzheimer's Disease

*You Jung Kang, Seung Jae Hyeon, Amanda McQuade, Jiwoon Lim, Seung Hyun Baek, Yen N. Diep, Khanh V. Do, Yeji Jeon, Dong-Gyu Jo, C. Justin Lee, Mathew Blurton-Jones, Hoon Ryu and Hansang Cho**

Supporting Information

Neurotoxic microglial activation via IFNγ-induced Nrf2 reduction exacerbating Alzheimer's disease

*You Jung Kang, Seung Jae Hyeon, Amanda McQuade, Jiwoon Lim, Seung Hyun Baek, Yen N. Diep, Khanh V. Do, Yeji Jeon, Dong-Gyu Jo, C. Justin Lee, Mathew Blurton-Jones, Hoon Ryu, and Hansang Cho****

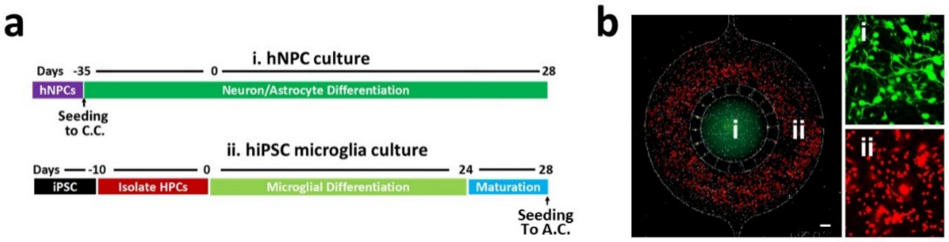

Figure S1. Timeline for the preparation of 3D human AD mini-brains

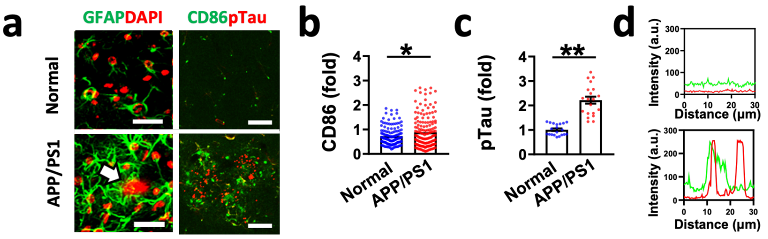

Figure S2. Characterization of microglia in brain tissues of APP/PS1 mice. a. Hippocampal CA1 region of brain tissue showing Aβ plaques (marked with arrows), reactive astrocytes ($GFAP^{high}$), and neurodegenerative microglia ($CD86^{high}$). **b-c.** Quantitative analysis confirming the significant increase of both **(b)** CD86 (two-tailed unpaired *t*-test, n=153) and **(c)** pTau (two-tailed unpaired *t*-test, n=20) in APP/PS1 mice compared to Normal. **d.** Co-localization of $CD86^{+}$ (marked in green) microglia with pTau region (red). Scale bars, 50 μm. All data represent means ± SD. *, P<0.05; **, P<0.01.

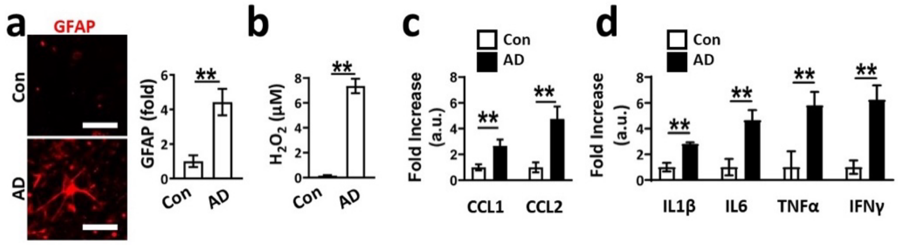

Figure S3. Induction of reactive astrocytes in 9 wks AD mini-brains (Neurons+Astrocytes) producing oxidative stress and proinflammatory cytokines. a. Immunostaining and quantitative results show the induction of reactive astrocytes ($GFAP^{high}$, red) in 9 wks AD mini-brains (two-tailed unpaired *t*-test, n=30). **b-d.** Production of **(c)** H_2O_2, **(d)** chemokines, and **(e)** proinflammatory cytokines by 9 wk AD mini-brains (one-way ANOVA with Tukey's multiple-comparisons test, n=5). Scale bars, 20 μm. All data represent means ± SD. *, $P<0.05$; **, $P<0.01$.

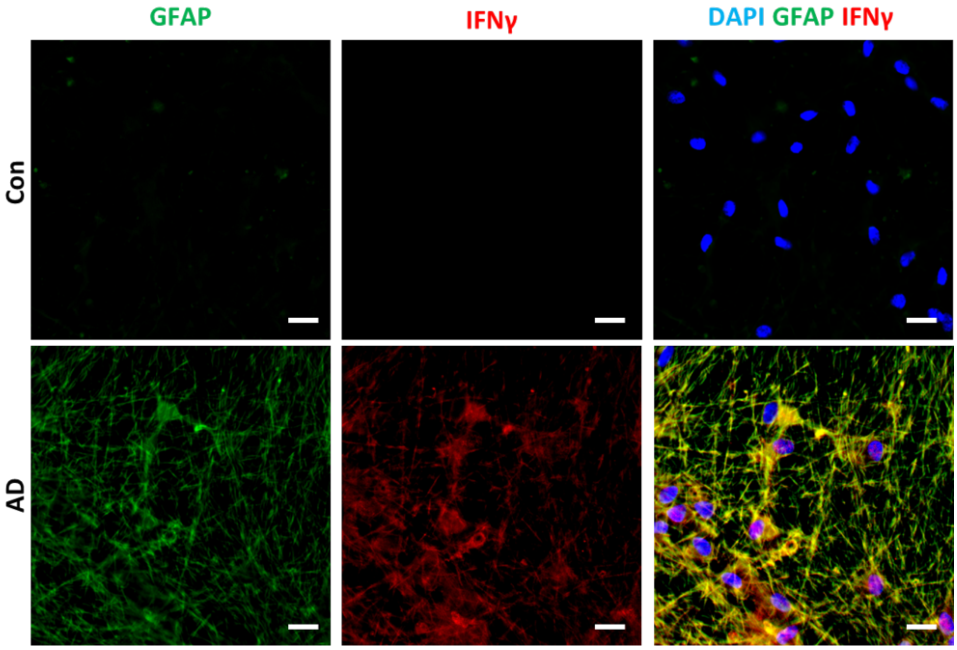

Figure S4. Production of IFNγ by reactive astrocytes in AD mini-brains. Immunostaining results show that the co-localization of reactive astrocytes (GFAP^{high}, green) and IFNγ (red) in 9 wks AD models. Scale bars, 20 μm.

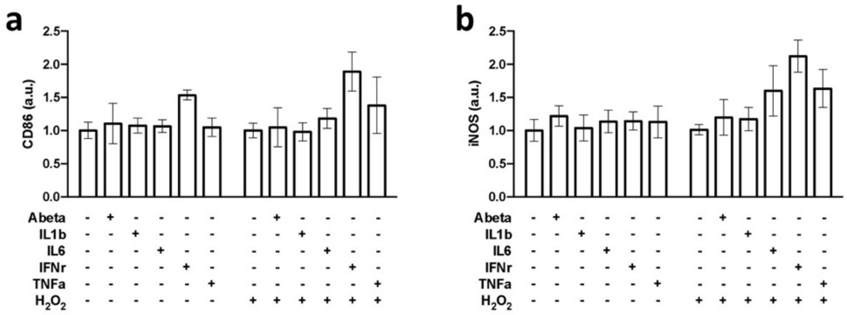

Figure S5. Investigation of soluble factors from human AD mini-brains affecting neurodegenerative phenotype transition. a-b. iMGs were treated with each soluble factor from 9 wks AD models and stained with neurodegenerative markers as **(a)** CD86 and **(b)** iNOS. All data represent mean ± SD (one-way ANOVA with Tukey's multiple-comparisons test, n=20).

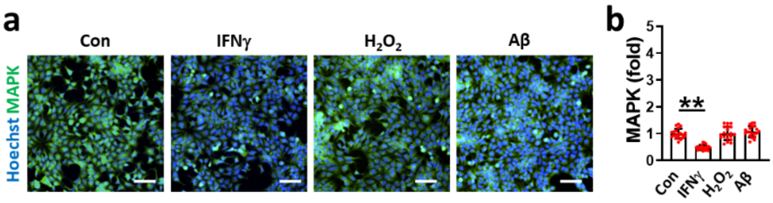

Figure S6. Investigation of MAPK changes in MG by soluble factors found in AD CM. a-b. (a) Florescent images and **(b)** quantitative results showing the expression of MAPK in MG treated with IFNγ (10 ng mL^{-1}), Aβ (100 ng mL^{-1} of Aβ40 and 10 pg mL^{-1} of Aβ42), or H_2O_2 (10 μM) (one-way ANOVA with Tukey's multiple-comparisons test, n=20). Scale bars, 50 μm. All data represent means ± SD. **, *P*<0.01.

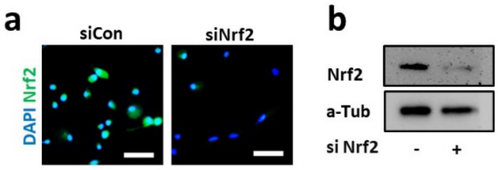

Figure S7. Confirmation of Nrf2 downregulation by siRNA. a-b. (a) Immunostaining and **(b)** western-blotting data confirmed the Nrf2 downregulation by the treatment of siNrf2 encapsulated in polymeric-based carriers. Scale bars, 20 μm.

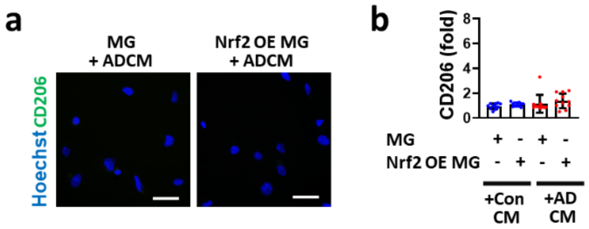

Figure S8. Investigation of neuroprotective phenotype transition by the treatment of AD CM. a-b. (a) Florescent images and **(b)** quantitative results showing the expression of CD206, a marker for M2 type microglia, in MG or Nrf2 OE MG treated with Con CM or AD CM (one-way ANOVA with Tukey's multiple-comparisons test, n=11). Scale bars, 50 µm. All data represent means ± SD.

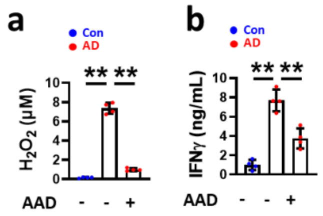

Figure S9. Effects of H_2O_2 scavenger on the production levels of H2O2 and IFNγ in AD mini-brains. a-b. (a) H_2O_2 and **(b)** IFNγ presented in the conditioned media of Con and AD mini-brains were measured (one-way ANOVA with Tukey's multiple-comparisons test, n=4). All data represent means ± SD. **, $P<0.01$.

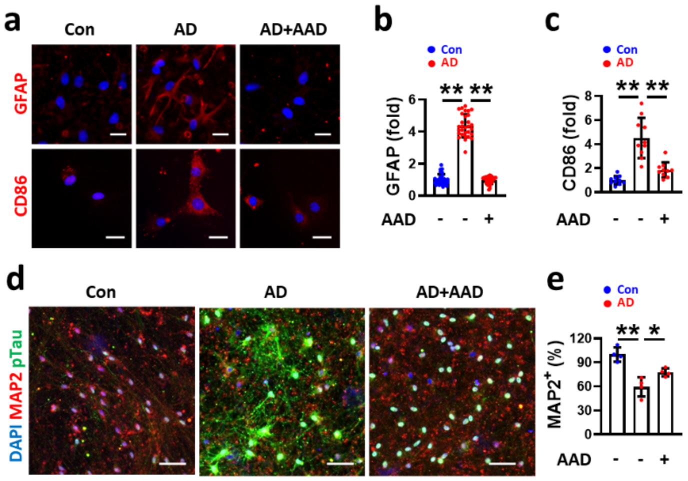

Figure S10. Effects of H_2O_2 scavenger on the glial activity and neurodegeneration. a. Fluorescent images showing the markers for astrocyte reactivity (GFAP) and neurodegenerative microglia (CD86). **b-c.** Quantitative analysis confirming the reduced population for **(b)** reactive astrocytes (one-way ANOVA with Tukey's multiple-comparisons test, n=30) and **(c)** neurodegenerative microglia in AD mini-brains by H_2O_2 scavenger (AAD) (one-way ANOVA with Tukey's multiple-comparisons test, n=10). **d.** Immunostaining results validating the reduction of pTau accumulation in AD mini-brains by AAD. **e.** Quantitative analysis confirming the recovery of neural populations by AAD (one-way ANOVA with Tukey's multiple-comparisons test, n=4). Scale bars, (a) 20 µm, (d) 100 µm. All data represent means ± SD. *, *P<0.05;* **, *P*<0.01.

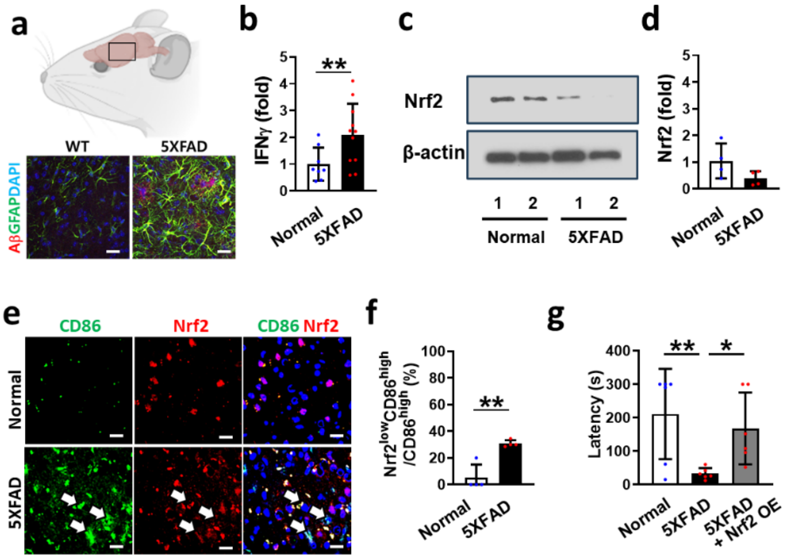

Figure S11. Downregulation of Nrf2 in neurodegenerative microglia correlates with neurodegeneration in AD animal models. a. Preparation of 5XFAD mouse models as described previously.[1] We assigned female and male mice (40-weeks old) to each group equally. Aβ plaques were formed in the cerebral cortex region of 5XFAD mice confirmed by immunostaining. **b.** Significant increase of IFNγ level was confirmed by qPCR (two-tailed unpaired *t*-test, n=11). **c-d. (c)** Western-blotting data and **(d)** quantitative analysis showing the expression level of Nrf2 in microglia extracted from Normal and 5XFAD mouse brains (two-tailed unpaired *t*-test, n=4). **e.** Immunofluorescent images of mouse brain tissues indicating the presence of $Nrf2^{low}CD86^{high}$ microglia (marked with arrows). **e-f. (e)** Fluorescent images and **(f)** quantitative analysis showing the significant increase of $Nrf2^{low}CD86^{high}$ population among the neurodegenerative microglia in microglia in 5XFAD mice (two-tailed unpaired *t*-test, n=4). **g.** Passive avoidance test confirming the recovery of fear conditioning memory by the overexpression of microglial Nrf2 in 5XFAD mice (one-way ANOVA with Tukey's multiple-comparisons test, n=6). Scale bars, 50 μm. All data represent means ± SD. *, $P<0.05$; **, $P<0.01$.

[1] H. Oakley, S. L. Cole, S. Logan, E. Maus, P. Shao, J. Craft, A. Guillozet-Bongaarts, M. Ohno, J. Disterhoft, L. V. Eldik, R. Berry, R. Vassar, *The Journal of Neuroscience*, **2006**, *26*, 10129.

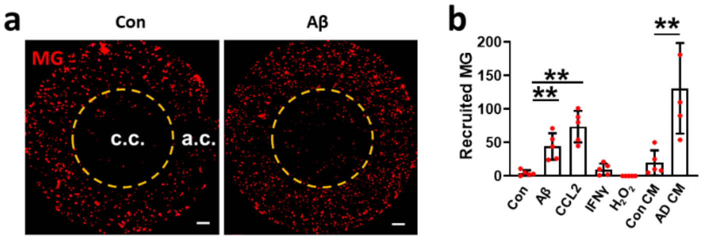

Figure S12. Induction of microglial recruitment by soluble factors from AD CM. a-b. (a) Fluorescent images and **(b)** quantitative analysis showing the microglial recruitment from the annular chamber (a.c.) to the central chamber (c.c.) by soluble factors found in AD CM (one-way ANOVA with Tukey's multiple-comparisons test, n=5). Scale bars, 500 μm. All data represents means ± SD. **, *P*<0.01.

Table S1. Information (Age, Sex, and Braak stage) of brain tissues from normal subjects and AD patients.

Number	Case	Age	Sex	Braak stage
1	Normal	86	M	II
2	Normal	87	F	II
3	Normal	89	M	II
4	Normal	67	M	I
5	Normal	82	M	I
6	Normal	83	M	I
7	mAD	71	M	I
8	mAD	92	F	II
9	mAD	86	M	III
10	mAD	87	M	III
11	mAD	93	F	II
12	mAD	88	F	II
13	sAD	88	F	VI
14	sAD	86	M	VI
15	sAD	94	F	V
16	sAD	66	M	VI
17	sAD	68	M	VI
18	sAD	91	F	V

Table S2. Antibodies used for this study

Antibodies	Company	Catalog #	Dilution ratio
GFAP	Sigma-Aldrich	AB5541	1:200 (Human)<br>1:500 (Mouse)
NeuN	Abcam	Ab177487	1:100
CD11b	Sigma-Aldrich	MABF515	1:100
CD86	Abcam	Ab119857	1:200
iNOS	Thermo Fisher Scientific	PA1-036	1:100
Nrf2	Santa Cruz	Sc-365949	1:100 (Human)
Nrf2	Abcam	Ab62352	1:200 (Mouse)
Keap1	Cell signaling	D6B12	1:100
MAPK	Cell signaling	9212S	1:100
pNFκB-p65	Cell signaling	6956	1:100
CD206	Novus Biologicals	NB6001415	1:100
Tuj1	BioLegend	801210	1:100
Synapsin-1	Abcam	Ab254349	1:100
pTau (AT8)	Thermo Fisher Scientific	MN1020	1:100 (Human)<br>1:200 (Mouse)
TREM2	R&D System	AF1828	1:100
Goat anti-chicken 2' ab	R&D System	NL017	1:200
Goat anti-mouse 2' ab<br>Alexa 488<br>Alexa 555<br>Alexa 647	Abcam	A32723<br>A32727<br>A32728	1:200
Goat anti-rabbit 2' ab<br>Alexa 488<br>Alexa 555<br>Alexa 647	Abcam	A32731<br>A32732<br>A32733	1:200
Donkey anti-chicken 2' ab<br>Alexa 594	Jackson	703-585-155	1:200
Donkey anti-mouse 2' ab<br>Alexa 647	Jackson	716-605-150	1:200
Donkey anti-rabbit 2' ab<br>Alexa 594	Jackson	711-585-152	1:200
Donkey anti-rat 2' ab<br>Alexa 488	Jackson	712-545-153	1:200

Table S3. Summary of statistical analysis

	Comparison	Sample number	Method(1)	P value	Significance(2)
Fig. 1d	Aβ40: Con+iMG vs AD+iMG	3	Two-tailed t test	<0.001	Y
	Aβ42: Con+iMG vs AD+iMG	3	Two-tailed t test	<0.001	Y
Fig. 1e	H_2O_2: Con vs AD	4	One-way ANOVA	<0.001	Y
Fig. 1f	IFNγ: Con vs AD	5	One-way ANOVA	<0.001	Y
Fig. 1g	Con vs Con+iMG	5	One-way ANOVA	0.027	Y
	Con vs AD	5	One-way ANOVA	0.010	Y
	Con vs AD+iMG	5	One-way ANOVA	<0.001	Y
	Con+iMG vs AD	5	One-way ANOVA	<0.001	Y
	Con+iMG vs AD+iMG	5	One-way ANOVA	<0.001	Y
	AD vs AD+iMG	5	One-way ANOVA	<0.001	Y
Fig. 1h	Con vs AD+iMG	7	One-way ANOVA	<0.001	Y
	Con+iMG vs AD+iMG	7	One-way ANOVA	<0.001	Y
	AD vs AD+iMG	7	One-way ANOVA	<0.001	Y
Fig. 1i	Con vs AD	4	One-way ANOVA	0.026	Y
	Con vs AD+iMG	4	One-way ANOVA	<0.001	Y
	AD vs AD+iMG	4	One-way ANOVA	0.042	Y
Fig. 1j	Normal vs sAD	4	Two-tailed t test	<0.001	Y
Fig. 2d	Con vs AD	3	Two-tailed t test	0.0054	Y
Fig. 2e	Con vs AD	8	Two-tailed t test	<0.001	Y
Fig. 2h	Control vs H_2O_2+ IFNγ	3	Two-tailed t test	0.0012	Y
Fig. 2i	Control vs H_2O_2+ IFNγ	8	Two-tailed t test	<0.001	Y
Fig. 3b	Con CM vs AD CM	16	Two-tailed t test	<0.001	Y
Fig. 3c	Con CM vs AD CM	8	Two-tailed t test	<0.001	Y
Fig. 3e	Con vs H_2O_2+IFNγ	15	One-way ANOVA	<0.001	Y
	H_2O_2 vs H_2O_2+IFNγ	15	One-way ANOVA	<0.001	Y
	IFNγ vs H_2O_2+IFNγ	15	One-way ANOVA	<0.001	Y
Fig. 3g	Con vs H_2O_2+IFNγ	20	Two-tailed t test	<0.001	Y
Fig. 3h	Con vs H_2O_2+IFNγ	30	Two-tailed t test	<0.001	Y
Fig. 3j	Con vs H_2O_2	20	One-way ANOVA	<0.001	Y
	H_2O_2 vs H_2O_2+IFNγ	20	One-way ANOVA	<0.001	Y
Fig. 3k	Con vs H_2O_2	8	One-way ANOVA	0.0011	Y
	H_2O_2 vs H_2O_2+IFNγ	8	One-way ANOVA	0.004	Y
Fig. 3m	Con CM vs AD CM	20	One-way ANOVA	<0.001	Y
	AD CM vs AD CM+4-OI	20	One-way ANOVA	<0.001	Y
Fig. 3n	Con CM vs AD CM	30	One-way ANOVA	<0.001	Y
	AD CM vs AD CM+4-OI	30	One-way ANOVA	<0.001	Y
Fig. 4b	siCon vs siCon+H_2O_2+IFNγ	7	One-way ANOVA	<0.001	Y
	siCon vs siNrf2+H_2O_2+IFNγ	7	One-way ANOVA	<0.001	Y
Fig. 4c	siCon vs siCon+H_2O_2+IFNγ	7	One-way ANOVA	<0.001	Y
	siCon vs siNrf2+H_2O_2	7	One-way ANOVA	<0.001	Y
Fig. 4d	siCon vs siCon+H_2O_2+IFNγ	7	One-way ANOVA	0.007	Y
	siCon vs siNrf2+H_2O_2	7	One-way ANOVA	<0.001	Y
Fig. 4f	AD CM+WT MG vs AD CM+Nrf2 OE MG	11	One-way ANOVA	<0.001	Y
	AD CM+WT MG vs AD CM+Nrf2 KD MG	11	One-way ANOVA	<0.001	Y
Fig. 4h	AD+WT MG vs AD+Nrf2 OE MG	12	One-way ANOVA	<0.001	Y
	AD+Nrf2 KD MG vs AD+Nrf2 OE MG	12	One-way ANOVA	<0.001	Y
Fig. 4j	AD+WT MG vs AD+Nrf2 OE MG	10	One-way ANOVA	<0.001	Y
	AD+Nrf2 KD MG vs AD+Nrf2 OE MG	10	One-way ANOVA	<0.001	Y
Fig. 5b	Con vs AD	10	Two-tailed t test	<0.001	Y
	Normal vs sAD	4	One-way ANOVA	0.0477	Y
	mAD vs sAD	4	One-way ANOVA	0.0015	Y
Fig. 5d	Normal vs sAD	4	Two-tailed t test	0.0183	Y
Fig. 5e	Normal vs sAD	40	Two-tailed t test	<0.001	Y

(1) One-way ANOVA was performed with Tukey post-hoc correction for multiple comparisons.
Two-tailed unpaired Student's t-test was performed for two variances.
(2) P value < 0.05 represents significance.
